# A Sustainable Biomineralization Approach for the Synthesis of Highly Fluorescent Ultra-Small Pt Nanoclusters

**DOI:** 10.3390/bios9040128

**Published:** 2019-10-29

**Authors:** Rajkamal Balu, Robert Knott, Christopher M. Elvin, Anita J. Hill, Namita R. Choudhury, Naba K. Dutta

**Affiliations:** 1 Chemical and Environment Engineering, School of Engineering, RMIT University, Melbourne, VIC 3000, Australia; 2 Australian Centre for Neutron Scattering (ACNS), Australian Nuclear Science and Technology Organisation (ANSTO), Lucas Heights, NSW 2234, Australia; 3 CSIRO Agriculture, Level 6, Queensland Bioscience Precinct, St Lucia, QLD 4067, Australia; 4 CSIRO Manufacturing, Bayview Ave, Clayton, VIC 3168, Australia

**Keywords:** quantum dot, noble metal clusters, fluorescent platinum nanoclusters, biosensors, biomineralization, intrinsically disordered protein, protein polymer, small angle X-ray scattering

## Abstract

Herein we report the first example of a facile biomineralization process to produce ultra-small-sized highly fluorescent aqueous dispersions of platinum noble metal quantum clusters (Pt-NMQCs) using a multi-stimulus responsive, biomimetic intrinsically disordered protein (IDP), Rec1-resilin. We demonstrate that Rec1-resilin acts concurrently as the host, reducing agent, and stabilizer of the blue-green fluorescent Pt-NMQCs once they are being formed. The photophysical properties, quantum yield, and fluorescence lifetime measurements of the synthesized Pt-NMQCs were examined using UV-Vis and fluorescence spectroscopy. The oxidation state of the Pt-NMQCs was quantitatively analyzed using X-ray photoelectron spectroscopy. Both a small angle X-ray scattering technique and a modeling approach have been attempted to present a detailed understanding of the structure and conformational dynamics of Rec1-resilin as an IDP during the formation of the Pt-NMQCs. It has been demonstrated that the green fluorescent Pt-NMQCs exhibit a high quantum yield of ~7.0% and a lifetime of ~9.5 ns in aqueous media. The change in photoluminescence properties due to the inter-dot interactions between proximal dots and aggregation of the Pt-NMQCs by evaporation was also measured spectroscopically and discussed.

## 1. Introduction

Metal nanoclusters (MNCs), comprised of only a few to a few tens of metal atoms, bridge the evolution of properties of isolated atoms to nanoparticles (NPs). They have the potential to exhibit unusual chemical, optical, electronic, and physical properties, which are drastically different from those of the bulk metal, NPs, or of the atom itself [1]. Pecision sized metal clusters synthesized in the gas phase have played a unique role in the areas of catalysis, energy research, and basic physical sciences, where the non-interacting clusters have been efficient in providing a fundamental understanding of cluster properties [2,3]. Surprisingly, noble metal nanoclusters (NMNCs) with sizes (generally <2 nm) comparable to the Fermi wavelength of an electron exhibit fluorescence with an emission wavelength correlated to the number of atoms in the cluster [4]. These fluorescence emitting NMNCs are often referred to as noble metal quantum clusters (NMQCs) for their discrete electronic state, which is similar to that of semiconductor quantum dots (QDs) [5]. NMQCs can be designed to possess high electron density, large Stokes shift, chemical stability, biocompatibility, catalytic activity, electrocatalysis, and excellent photostability relative to other base metals, which make them the promising candidates for creating highly polarizable molecular scale metal QDs with strong optical responses in aqueous media [6,7]. They are an emerging area of nanoscience and are of significant interest in their optimization for a variety of applications in fluorescence detection, electroluminescent display, solid-state lighting, photovoltaics, catalysis, bio-markers, and biomedical applications [8,9,10,11]. However, the synthesis of such NMQCs of atomically precise nanoclusters represents several unique synthetic and characterization challenges, e.g., the employment of tedious and complex synthesis processes, use of harsh reaction conditions, and utilization of organic solvent that may hinder their future development for biomedical applications [12]. They are also infamously low yielding and often generate complicated mixtures. Extensive research has been carried out on gold and silver NMQCs. However, reports on the successful synthesis of platinum NMQCs (Pt-NMQCs) are scarce, and only a few reports on successful synthesis using thiol, dendrimer, and polymer have been demonstrated [13].

Over the last decades or so significant progress has been made in the use of a supramolecular approach for the preparation of engineered NPs [14,15]. The use of plant extracts in the reduction and stabilization of metal ions has attracted significant recent attention as a green route for the synthesis of metal NPs [16] and DNA assembled metal nanoclusters (NCs). Promisingly, environmentally benign approaches to construct nanoclusters through DNA-based conjugation and modulation have also been advanced [17]. Inspired by biomineralization in nature, recently, protein/peptide mediated syntheses of inorganic NPs have been identified as a promising strategy. The mineral formation strategy in biology is unique, occurs at physiological conditions, and the products of this process are diverse. Biomineralization not only has the potential to form incredible hierarchical structure but also can generate elegant graded structure. Depending on the unique primary sequence, sequence-specific 3D structure, environmental-responsiveness, self-assembly characteristics and reaction micro-environment (e.g., pH, temperature, reactant concentration), the production of a variety of noble metal NPs of different size, shape, and morphology has been reported [18,19,20,21,22]. However, the peptide sequence–activity correlation, sequence–crystal growth relationship, and sequence–stabilization capability for the NPs formed are complex and not yet clearly understood [23,24,25]. Therefore, dialing different amino acid sequences and the reaction microenvironment for the synthesis of designer NMQCs of controlled shape, size, and activity are still illusive. Noble metal NCs/NPs stabilization by proteins has so far been demonstrated mainly using globular proteins with sulfur-containing cysteine and methionine amino acid residues. They stabilize the nucleated cluster through broken disulphide bonds [26,27]. Interestingly, it has been established through extensive research that in both vertebrates and invertebrates, intrinsically disordered proteins (IDPs) play a controlling role in the biomineralization process; and indeed, all the proteins associated with mineralization in the Swiss Protein Database are IDPs [28]. The incorporation of inorganic materials into IDP domains and generating controlled crystals is not trivial, and only through the control of electrostatic, dipolar, hydrogen bonding, and complexation interactions can inorganic materials be incorporated/directed in a pre-determined fashion. Here, we propose an innovative green synthesis approach for the synthesis of Pt-NMQCs using a simple, scalable, and benign biomineralization process using a designer IDP, Rec1-resilin, which contains no cysteine or methionine residues. We demonstrate that Rec1-resilin acts concurrently as the host/structure-directing agent, the reducing agent, and the stabilizer of NCs once they are being formed, and the resulting NCs are highly fluorescent.

Native resilin is an elastomeric insect protein reported for outstanding resilience (>92%) and a fatigue life in excess of 300 million cycles. Rec1-resilin, the first resilin-mimetic protein, was derived from the N-terminal elastic repeat domain (Exon 1) of the fruit fly *Drosophila melanogaster* resilin gene and expressed as a water-soluble protein in *Escherichia coli* [29]. Recently, we reported the unusual multi-stimuli responsiveness of Rec1-resilin in aqueous solution [30] and demonstrated its use in creating patterned surfaces and responsive interfaces [31]. Mayavan et al. [32] demonstrated a one-step protocol to synthesize nanobioconjugates containing gold NPs and Rec1-resilin. Recently, Dutta et al. [33,34] reported the synthesis of size-controlled platinum NPs using Rec1-resilin as a soft template and its effectiveness as a fuel cell electrocatalyst. In this work, we not only demonstrate successful synthesis of Pt-NMQCs using Rec1-resilin as a directing agent but also attempt to present a very high level of understanding of the structure and conformational dynamics of IDPs during the formation of Pt-NMQCs that focuses on the critical issues of the role of an IDP in biomineralization.

## 2. Materials and Methods

### 2.1. Protein Expression and Purification

Rec1-resilin was synthesized by a cloning technique, as reported previously [29]. Briefly, exon-1 of the *Drosophila melanogaster* CG15920 gene (19-321 of a 620-amino acid sequence from the N-terminal region) was cloned and expressed in the bacteria *Escherichia coli* as a water-soluble protein. The purification of synthesized protein was performed using a three-step procedure: (1) salt precipitation using 20% ammonium sulphate, (2) overnight dialysis in excess phosphate-buffered saline (PBS) at 4 °C, and (3) stirring and heating at 80 °C for 10 min. The denatured proteins were then removed by centrifugation at 12,000 rpm for 15 min, and the resulting supernatant was freeze dried and stored at −20 °C. The synthesized Rec1-resilin consisted of 310 amino acid residues with 18 copies of a 15-residue repeat sequence, GGRPSDSYGAPGGGN, as shown in Figure S1 in the Supporting Information.

### 2.2. Molecular Weight Determination by Matrix Assisted Laser Desorption Ionization Time-of-Flight (MALDI-TOF) Mass Spectrometry

The molecular weight of synthesized Rec1-resilin was determined using an ultrafleXtreme MALDI-TOF mass spectrometer (Bruker Daltonics, Hamburg, Germany). Briefly, 3 mg of synthesized Rec1-resilin was dissolved in 30% acetonitrile with 0.1% trifluoroacetic acid (TA30) solution to obtain a final protein concentration of 3.0 mg/mL. A total of 1.5 µL of the saturated sinapinic acid matrix was spotted onto a polished steel target plate and air dried. Then, 2 µL of prepared protein solution (1 pmol/µL) was mixed with equal volumes of matrix solution, and 0.5 µL of the mixture was spotted onto the previously created matrix spots and air dried. Mass spectra were acquired in the measurement range of 5000–50,000 kDa. A total of 5000 shots were collected for the external calibration, and 20,000 shots for sample measurement. The obtained mass spectra (Figure S2 in the Supporting Information) were analyzed using Bruker Daltonics flexAnalysis software employing background subtraction, smoothing, and peak detection algorithms. A molecular weight of 28.5 kDa was obtained for the synthesized Rec1-resilin.

### 2.3. Synthesis of Fluorescent Pt-NMQCs

**Figure 1:**
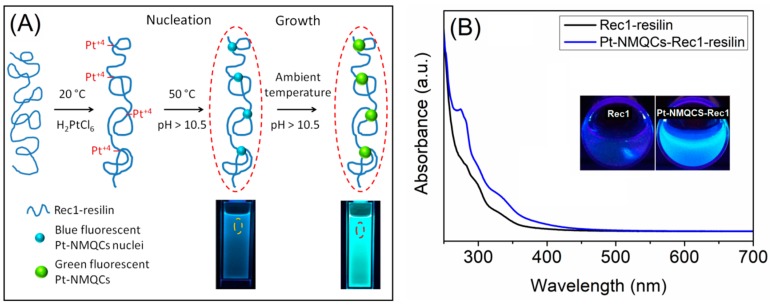
(**A**) Schematic representation of Rec1-resilin-directed ‘green synthesis’ of the fluorescent platinum noble metal quantum clusters (Pt-NMQCs) employed. (**B**) UV-visible absorption spectra of pristine Rec1-resilin (at pH 12) and blue fluorescent Pt-NMQCs-Rec1-resilin nanobioconjugates. Insets are the corresponding optical images of samples under 365 nm UV light.

In this work, the preparation of fluorescent Pt-NMQCs-Rec1-resilin nanobioconjugates was carried out using a one-pot green synthesis approach, where the pH-responsiveness of Rec1-resilin was tuned to reduce and stabilize Pt-NMQCs, as shown in Figure 1A. Briefly, 0.5 mL of 1 M sodium hydroxide solution was added to 8.25 mL of 0.6 wt.% Rec1-resilin solution (prepared in 10 mM PBS) to bring the solution pH to ~12. The protein solution was incubated at 25 °C for 5 min to trigger the intrinsic redox potential of the protein, where tyrosine (Tyr) amino acid residue (pKa ~10.5) in Rec1-resilin de-protonated to tyrosinate at pH ≥ 10.5, thereby generating electrons for metal ion reduction [19,22]. Aqueous metallic precursor solution (6 mM hexachloroplatinic acid) was then added to the above protein solution, vortex mixed for 5 min, and incubated in the dark (as Tyr amino acid residue is light sensitive) without disturbing for 10 days at 50 °C to initiate Pt-NMQCs nucleation. The Pt-NMQCs-Rec1-resilin nanobioconjugates containing nucleated blue fluorescent Pt-NMQCs were then incubated for eight weeks at ambient temperature for controlled growth of green fluorescent PtNMQCs.

### 2.4. Photophysical Properties, Concentration, and Oxidation State of Pt-NMQCs

The photophysical properties of pristine Rec1-resilin and Pt-NMQCs-Rec1-resilin nanobioconjugates were examined by UV-Vis and fluorescence spectroscopy. UV-Vis absorption spectra of the samples were recorded using an Evolution 201 UV-Vis spectrophotometer (Thermo Scientific Australia Pty Ltd., Melbourne, Australia), whereas steady state fluorescence excitation and emission measurements were recorded using a Cary Eclipse fluorescence spectrophotometer (Varian Inc., Palo Alto, CA, USA). The quantum yield and lifetime measurements of green fluorescent Pt-NMQCs-Rec1-resilin nanobioconjugates were recorded using an FLS980 fluorescence spectrophotometer (Edinburgh Instruments Ltd., Livingston, UK). The concentration of Pt in blue fluorescent Pt-NMQCs-Rec1-resilin nanobioconjugate dispersion was measured using an Optima 5300DV inductively coupled plasma optical emission spectrometer, ICP-OES (PerkinElmer, Melbourne, Australia). The chemical states of the synthesized Pt-NMQCs were examined by X-ray photoelectron spectroscopy (XPS). XPS spectra of Pt-NMQCs-Rec1-resilin nanobioconjugates were recorded using an AXIS Ultra delay-line detector X-ray photoelectron spectrometer (Kratos Analytical Ltd., Manchester, UK), and the binding energies of all peaks were referenced to a C1s value of 284.6 eV.

### 2.5. Small Angle X-ray Scattering (SAXS)

The structural information of pristine Rec1-resilin and blue fluorescent Pt-NMQCs-Rec1-resilin nanobioconjugates was investigated using a bench-top Bruker NanoSTAR II SAXS equipped with a rotating anode Cu Kα radiation source. The X-ray scattering angle and intensities were recorded using a 2D detector placed at 90° to the incident X-ray beam. A scattering vector, q (Equation (1)) in the range of 0.012 to 0.39 Å^−1^ was used for the measurements and analysis:

q = 4πsinθ/λ,(1)

where θ is the angle of scattering and λ is the wavelength of X-rays (1.54 Å). The samples were placed in a quartz capillary with temperature controlled at 20 ± 0.1 °C, and the data were collected twice for an hour and averaged. No change in the scattering cross section of the blue fluorescent Pt-NMQCs-Rec1-resilin nanobioconjugates (Figure S3 in the Supporting Information) over a period of two hours suggests no radiation damage and structural stability of the system with exposure to X-rays. In all the cases the instrument and buffer background were subtracted (using the respective sample T_SAS_ values) from the sample scattering using the PRIMUS computer program [35]. The size and conformation of the protein in samples were obtained using the Guinier approximation (Equation (2)) in the low-q region [36], Porod analysis in the high-q region [37], pair distance distribution function, P(r) and Kratky plot for the full-q region [38]:

I(q) = I(0) exp(−q^2^R_g_^2^/3); (qR_g_ ≤ 1.3), (2)

where I(q) is the intensity of scattering, I(0) is the intensity at zero scattering, and R_g_ is the radius of gyration of the protein. The background scattering from the sample was determined with a high-q power law fit (0.8 < q < 0.39) using the SasView computer program (https://www.sasview.org/) and subtracted from the data for analysis [34]. The structural parameters of Rec1-resilin were also determined by fitting the desired model functions to SAXS data using the SasView computer program.

## 3. Results and Discussion

### 3.1. Photophysical Properties and Oxidation State of Pt-NMQCs

**Figure 2:**
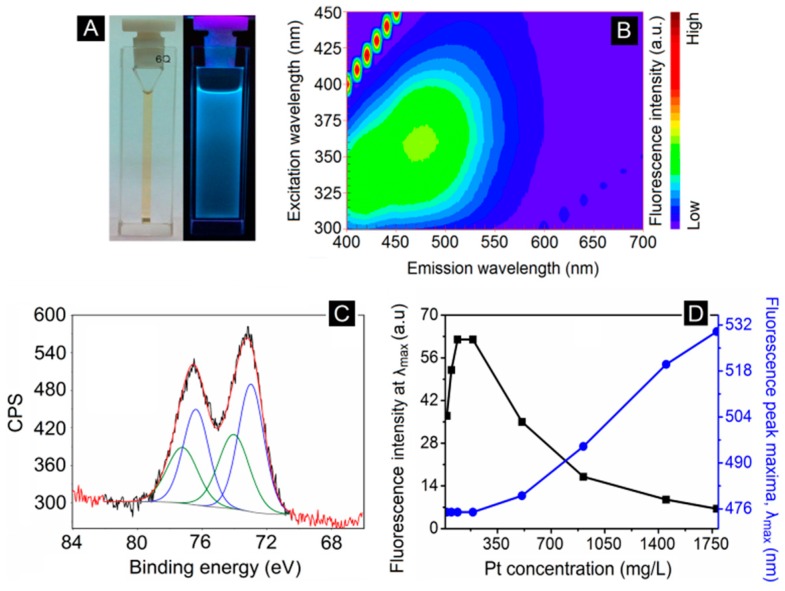
(**A**) Optical images under visible and 365 nm UV light, (**B**) 3D-fluorescence matrix contour plot, and (**C**) deconvoluted Pt 4f XPS spectrum (blue peaks for Pt^0^ and green for Pt^2+^) of the blue fluorescent Pt-NMQCs-Rec1-resilin nanobioconjugates. (**D**) Effect of Pt-NMQCs concentration on photoluminescence property of the blue fluorescent Pt-NMQCs-Rec1-resilin nanobioconjugates.

Figure 1B shows bright blue fluorescence (typically brighter than Rec1-resilin itself; under 365 nm UV light) from the equilibrated solution after successful reduction of platinum ions, indicating the formation of nucleated Pt-NMQCs. The UV-Vis spectra of pristine Rec1-resilin (at pH ~12) and as prepared blue fluorescent Pt-NMQCs-Rec1-resilin nanobioconjugates reveal the tyrosine (~275 nm), tyrosinate (~290 nm), dityrosine (~325 nm), and nucleated Pt-NMQCs (~375 nm) absorption peaks and plateau, as shown in Figure 1B. The as prepared blue fluorescent Pt-NMQCs-Rec1-resilin nanobioconjugate dispersion was then ultra-centrifuged at 10,000 rpm for 5 min to remove any large Pt-NPs formed. A Pt concentration of 145 mg/L was determined for the centrifuged supernatant using ICP-OES. Figure 2A shows the optical images of centrifuged supernatant under visible and 365 nm UV light. The 3D-fluorescence matrix contour plot of centrifuged supernatant displayed two concentric excitation/emission maxima (EEM) at ~360/475 nm and ~325/420, respectively (Figure 2B). Conversely, pristine Rec1-resilin solution showed single weak concentric EEM at ~325/420 nm for the same experimental conditions (Figure S4 in Supporting Information). Therefore, the EEM at ~360/475 and ~325/420 nm of the blue fluorescent Pt-NMQCs-Rec1-resilin nanobioconjugates are attributed to nucleated Pt-NMQCs and Rec1-resilins’s dityrosine fluorescence, respectively. The dityrosine cross-links in the protein could be formed by the oxidation of Tyr residues at pH > pKa of the Tyr [39]. The noble metal ion-induced fluorescence signal characteristic of Tyr cross-linking is also supported by previous reports [40].

Figure 2C represents the high-resolution sweeps of the Pt (4f) XPS spectrum of the dried blue fluorescent Pt-NMQC-Rec1-resilin nanobioconjugates and revealed the existence of both Pt(0) and Pt(II) valence state of Pt; where the data could not be fit with unimodel band peaks (Figure S5 in Supporting Information). The binding energy peak position of Pt(0) 4f7/2 was obtained at 72.5 eV, demonstrating a positive binding energy shift relative to that of bulk Pt (71.1 eV). Based on the literature reports, this shift may orginate from the well known cluster size effects in XPS giving rise to size-dependent binding energy shifts relative to bulk metal, and represents the formation of sub-nanometer clusters [41,42,43]. Moreover, in the absence of precursor, Pt(IV) valence state was also evident from the XPS spectrum. The best fit of the experimental curve revealed that the nucleated Pt-NMQC contained ~74% Pt(0) and ~26% Pt(II). The changes in the electronic structure of Pt-NMQCs have also been demonstrated elsewhere using other methods. Recently, Duchesne and Zhang [44] employed X-ray absorption spectroscopy to probe the local structure and electronic properties of a series of Pt-NMQCs synthesized and stabilized using *N*,*N*-dimethylformamide, and revealed an apparent lack of metallic Pt–Pt bonding in the samples examined, and proposed that a non-metallic Pt-NMQC was responsible for the observed fluorescence behavior. More insightful research is warranted to identify the exact local electronic structure of Pt-NMQCs. From the fluorescence peak maximum (~475 nm), the number of atoms in the nucleated Pt-NMQCs was estimated to be Pt5 (size < 1.5 nm [41]), using the spherical Jellium model [6,11] for the energy gap in Pt-NMQCs of E_Fermi_/N^1/3^; where E_Fermi_ is the Fermi energy of Pt (E_Fermi_ = 4.28 eV) and N is the number of Pt atoms in the cluster. However, based on the obtained XPS results and Jellium modeling, one would expect approximately four Pt(0) and one P(II) for a Pt5 cluster, which is very unlikely for a stable cluster. Therefore, the results indicate the presence of a mixture of clusters in blue fluorescent Pt-NMQCs-Rec1-resilin nanobioconjugate dispersion instead of one single cluster. 

### 3.2. Inter-Dot Distance Dependence of the Fluorescence Property of Blue Fluorescent Pt-NMQCs-Rec1-Resilin Nanobioconjugates

The inter-dot distance dependence of the photoluminescence has been reported in semiconductor QDs, and optical coupling between QDs is of great importance when the QD concentration in the actual device is increased [45]. In order to investigate the influence of inter-nucleated Pt-NMQCs distance on photoluminescence of Pt-NMQCs, the blue fluorescent Pt-NMQCs-Rec1-resilin nanobioconjugate dispersion was concentrated (by rota-vaporization) to yield a nanobioconjugate dispersion with 1780 mg/L of Pt (measured using ICP-OES). The concentrated dispersion was then diluted to 1450, 910, 510, 190, 90, 50, and 20 mg/L level in water and measured for the fluorescence EEM corresponding to nucleated Pt-NMQCs, dityrosine, and their respective fluorescence intensity and peak maxima. Figure 2D shows the effect of Pt-NMQCs concentration (or inter-dot distance) on the photoluminescence properties of the blue fluorescent Pt-NMQCs-Rec1-resilin nanobioconjugates. Interestingly, the 1780 mg/L of Pt-NMQCs containing nanobioconjugate dispersion exhibited red shift in fluorescence (~475 to ~530 nm) with a single concentric emission maximum, and a decreased fluorescence intensity (Figure S6 in Supporting Information). Conversely, with a decrease in Pt-NMQCs concentration in the nanobioconjugate dispersion, the fluorescence intensity of nucleated Pt-NMQCs increased progressively to reach a maximum in the range 90–190 mg/L, and subsequently decreased upon further decrease in Pt-NMQCs concentration. Also, an investigation of the influence of the Pt-NMQCs concentration on the dityrosine fluorescence property of the nanobioconjugates resembled that of nucleated Pt-NMQCs, with dityrosine fluorescence intensity reaching a maximum at 90 mg/L (Figure S6 in Supporting Information). The dityrosine fluorescence peak maximum did not change position with the dilution of Pt-NMQCs (Figure S7A in Supporting Information). Therefore, the effect of change in the fluorescence property of the nanobioconjugates is attributed to the inter-nucleated Pt-NMQCs distance. The concentration quenching may be related to the dipole–dipole (d–d) interaction between Pt-NMQCs. This result is also confirmed by the reversibility of fluorescence property (two concentric EEM at ~360/475 nm and ~325/420) of blue fluorescent Pt-NMQCs-Rec1-resilin nanobioconjugates with dilution (Figure S6 in Supporting Information). Furthermore, the change in nucleated Pt-NMQCs/dityrosine fluorescence intensity ratio clearly indicates that with increased Pt-NMQC concentration (i.e., decreased nucleated Pt-NMQCs distance) the fluorescence of dityrosine was quenched by nucleated Pt-NMQCs (Figure S7B in Supporting Information).

### 3.3. Change in Conformational Organization of Rec1-Resilin in Pt-NMQC-Rec1-Resilin Nanobioconjugates

**Figure 3:**
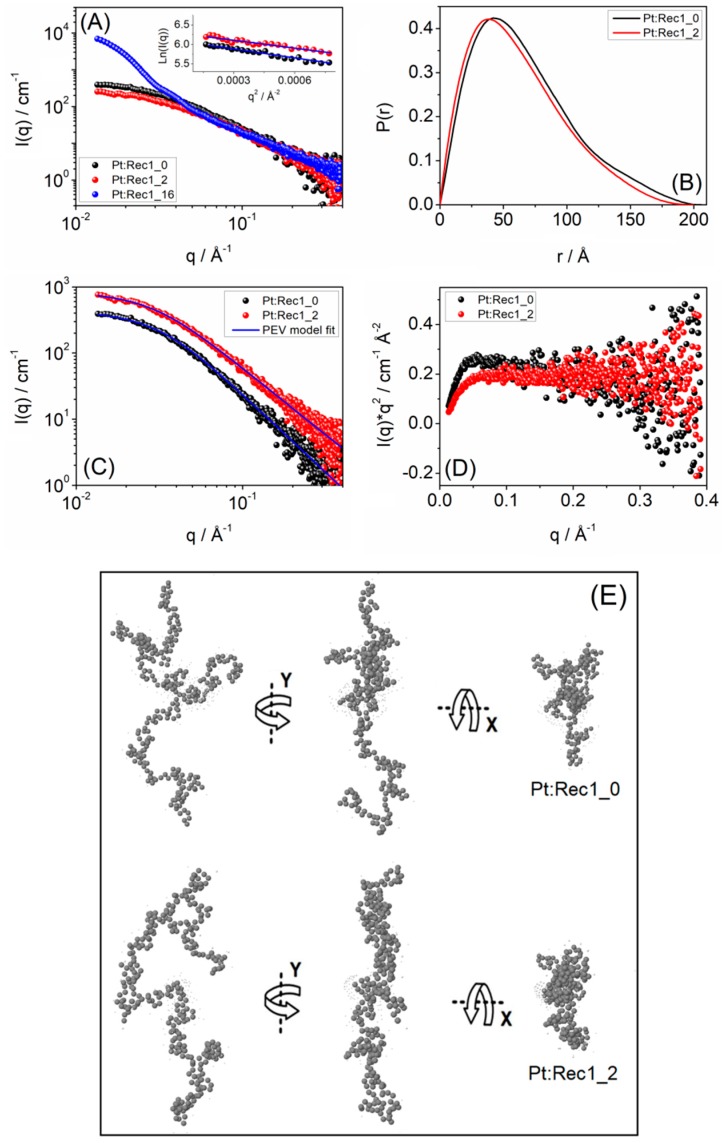
(**A**) Small angle X-ray scattering (SAXS) pattern in logarithmic scale (inset: Guinier plot), (**B**) pair distance distribution function, P(r) curve, (**C**) polymer excluded volume (PEV) model fit, (**D**) Kratky plot, and (**E**) representative ab initio 3D model structure (one among an infinite ensemble of possible 3D-densities reconstructed using the GASBOR computer program from the P(r) output) of pristine Rec1-resilin (Pt:Rec1_0) and blue fluorescent Pt-NMQCs-Rec1-resilin nanobioconjugates (Pt:Rec1_2).

Rec1-resilin is an IDP which is characterized by conformational heterogeneity and a lack of persistent secondary/tertiary structure, and represents a dynamic structural ensemble [46,47]. Consequently, it continues to be very difficult to investigate the structural heterogeneity and dynamical properties of IDPs, both experimentally and through simulation studies. To reveal the conformational organization and secondary structural changes of Rec1-resilin in detail before and after the formation of blue fluorescent PtNMQCs-Rec1-resilin nanobioconjugates, the samples were investigated using SAXS. SAXS is a powerful technique for determining the size, shape, and structure of the population-weighted average conformational ensemble of IDPs. Figure 3A shows typical SAXS intensity plots of the pristine Rec1-resilin solution (Pt:Rec1_0, i.e., Pt:Protein molar ratio of 0) and blue fluorescent Pt-NMQCs-Rec1-resilin nanobioconjugate dispersion (Pt:Rec1_2, i.e., Pt:Protein molar ratio of 2). The SAXS patterns of nanobioconjugates showed moderately increased scattering intensity only at higher q-regime over pure protein alone, indicating the formation of nucleated Pt-NMQCs (Figure 3A) [48]. However, the precise size and structure of blue fluorescent Pt-NMQCs could not be measured using SAXS, due to the limitation of the particle size measurement range (1.6–52.3 nm) of the instrument. With an increase in the Pt:Protein molar ratio from 2 to 16 (Pt:Rec1_16), the formation of larger Pt-NPs (>5 nm and non-fluorescent) was evident, with a dramatic increase in SAXS intensity at lower q-regime (Figure 3A). Pt-atoms are electron-rich and thus have electron density many-fold over that of protein. These observations indicate that an optimal Pt:Protein ratio is required for optimum Pt-NMQC formation. The Guinier analysis/approximation of the scattering data allowed for direct estimation of the R_g_ of the protein construct [49]. The Guinier plot is an algebraic transformation (ln(I) versus q^2^) of the data, which produces a linear q^2^ dependence in the “Guinier region” found at very small scattering angles (q < 0.05 Å^−1^). The slope of the data is directly proportional to the R_g_ of the overall protein chain (see inset, Figure 2A). The R_g_ values obtained from Guinier approximation were 50.5 ± 1.5 Å and 45.1 ± 1.3 Å, describing the overall equilibrium conformation of the pristine Rec1-resilin and Rec1-resilin in Pt-NMQCs-Rec1-resilin nanobioconjugates, respectively. However, Guinier’s law is reported to be less appropriate and often underestimates the R_g_ values of extended chain structures, such as IDPs [46,50].

On the other hand, the model independent pair distance distribution function (PDDF), P(r) is a descriptive way to elucidate particle/molecular distribution and provides information on the distribution of all inter-atomic distances (r), and not just the average distance, as obtained from the Guinier slope [38], and is therefore considered to be more appropriate for R_g_ calculations of IDPs than Guinier’s approximation [50]. The comparison of the P(r) from different conditions indeed revealed condition-specific conformational changes. Unlike the Guinier slope, R_g_ calculated from the P(r) distribution used all of the experimental data and was determined in real space R_g_. The P(r) distribution curve fit for both the pristine Rec1-resilin and blue fluorescent Pt-NMQC-Rec1-resilin nanobioconjugates displayed asymmetric elongated curves, which is a signature of unfolded proteins (Figure 3B) [38]. The real space R_g_ of the pristine Rec1-resilin and Rec1-resilin in PtNMQCs-Rec1-resilin nanobioconjugates were estimated to be ~54.2 Å (persistence length, ξ = 38.3 Å, R_g_ = √2ξ; end-to-end, R_1n_ = 132.8 Å, R_1n_ = √6R_g_) and ~51.0 Å (36.1 Å, 124.9 Å), respectively, using inverse Fourier transform from the scattering intensity, I(q). Also, the maximum molecular dimension (D_max_) of the Rec1-resilin in PtNMQCs-Rec1-resilin nanobioconjugates was estimated to be ~200.5 Å from the P(r) function, which is slightly less than that of pure Rec1-resilin (~205.5 Å). The obtained structural parameters of pristine Rec1-resilin are in general agreement with previous reports [46,47]. 

The R_g_ of pristine Rec1-resilin and nanobioconjugates obtained by P(r) fit was also validated using a shape-independent polymer excluded volume (PEV) model fit (Figure 3C) [51]. The model describes the scattering from polymer chains subject to excluded volume effects and can be used to estimate the R_g_ and fractal dimension of the scattering molecule. The PEV model fit returned R_g_ of ~53.8 Å and ~51.6 Å for the pristine Rec1-resilin and Rec1-resilin in blue fluorescent PtNMQCs-Rec1-resilin nanobioconjugates, respectively, which is consistent with P(r) fit results. The fit also returned a Porod exponent (m) value of 2.3 ± 0.1 (signature of partially collapsed Gaussian chain IDP) for the pure Rec1-resilin, and 2.0 ± 0.1 (signature of swollen Gaussian coil) for the nanobioconjugates. The Porod region corresponds to a probed range smaller than the scattering objects and the scattering radiation potentially examines the local structure. The observed conformational transformation of pure Rec1-resilin and nanobioconjugates was further assessed using a Kratky plot [50]. The Kratky plot describes the “unfoldedness” or “random coil” likeness of the scattering molecules, where the nanobioconjugates displayed a monotonic increase throughout q, whereas pristine Rec1-resilin displayed an initial monotonic increase in the lower q-region followed by a plateau in the higher q-region (Figure 3D). The observed trend supports the validation of Porod slope from PEV fit [36]. The in-depth analyses of the SAXS data set confirmed that after interaction and stabilization of Pt-NMQC, the overall intrinsic structure of Rec1-resilin remained intrinsically disordered, with reorganization of molecular conformation from a partially collapsed Gaussian chain IDP towards a swollen coil Gaussian chain IDP. Figure 3E shows a typical ab initio reconstruction (made using GASBOR computer program [52]) of pristine Rec1-resilin and nanobioconjugates from SAXS data. The 3D-model structure (one among an infinite ensemble of possible 3D-densities) is presented as a chain-like ensemble of dummy amino acid residues (310 numbers) placed anywhere in continuous space with a preferred number of close distance neighbors. A Chi square (χ^2^) value of 0.90, representing the goodness of fit, was obtained for reconstruction fits. 

### 3.4. Evolution of Nucleated Pt-NMQCs with Time

**Figure 4:**
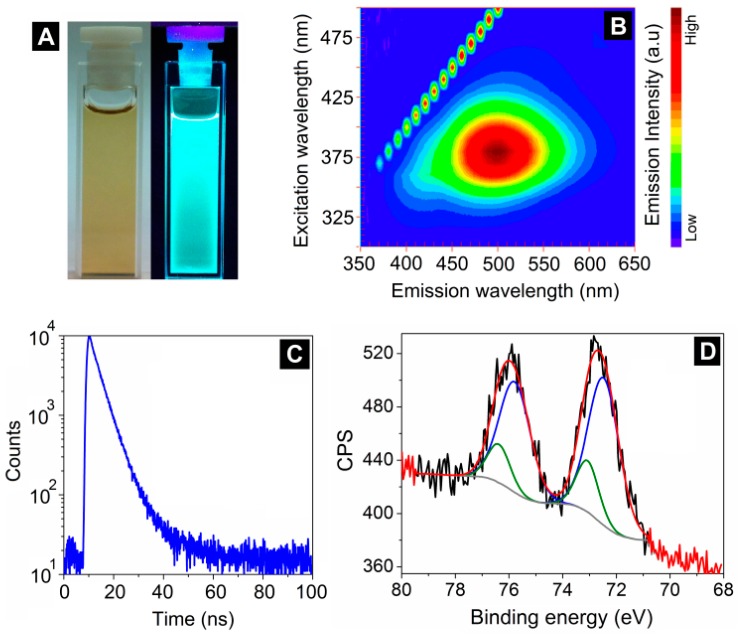
(**A**) Optical images under visible and 365 nm UV light, (**B**) 3D-fluorescence matrix contour plot, (**C**) fluorescence lifetime spectrum, and (**D**) deconvoluted Pt 4f XPS spectrum (blue peaks for Pt^0^ and green for Pt^2+^) of the green fluorescent Pt-NMQCs-Rec1-resilin nanobioconjugates.

The centrifuged blue fluorescent Pt-NMQCs-Rec1-resilin nanobioconjugate dispersion (145 mg/L of Pt) was incubated at room temperature (~20 °C) for a long period of time in the dark to study the evolution of nucleated Pt-NMQCs with time. The incubated supernatant developed green fluorescence after eight weeks (Figure 4A), indicating growth in the cluster size of the nucleated Pt-NMQCs. The 3D-fluorescence matrix contour plot of this green fluorescent Pt-NMQCs-Rec1-resilin nanobioconjugates displayed EEM at ~380/500 nm (Figure 4B), corresponding to Pt-NMQCs with a Stokes shift of 0.79 eV. The absolute quantum yield (Q_y_) of the generated green fluorescent Pt-NMQCs was calculated using Equation (3) [53]:

Q_y_ = P*N*_em_/P*N*_ab_, (3)

where P*N*_em_ and P*N*_ab_ are the number of emitted and absorbed photons by the fluorescent Pt-NMQCs, measured using a fluorescence spectrophotometer.

The Q_y_ of the synthesized green fluorescent Pt-NMQCs was measured as ~7.0% in water. The Pt-NMQCs-Rec1-resilin nanobioconjugates exhibited three radiative transition lifetimes (τ) 0.74 ± 0.05 ns, 3.77 ± 0.05 ns, and 9.48 ± 0.63 ns (Figure 4C), where the first two lifetimes could be associated with dityrosine [39]. The 4f7/2 XPS spectrum of green fluorescent Pt-NMQCs nanobioconjugates revealed the existence of both Pt(0) and Pt(II) valence states with the binding energy of Pt(0) observed at 72.4 eV (Figure 4D). The green fluorescent Pt-NMQCs showed an increase (from ~74% to ~80%) in Pt(0) valence state compared to the nucleated blue fluorescent Pt-NMQCs consistent with the growth of nucleated Pt-NMQCs. The luminescence of green fluorescent Pt-NMQCs-Rec1-resilin nanobioconjugate dispersion remained stable for almost a year (observed period), indicating no further growth in cluster size (Figure S8 in Supporting Information). The exceptional stability of the stabilized nanobioconjugates is related to the repulsive interaction among the entities due to the negative surface charge of the stabilizing agent, Rec1-resilin under the established environment.

## 4. Conclusions

In summary, we demonstrated a one-pot green synthesis method for the preparation of highly stable, blue and green fluorescent, water-soluble Pt-NMQCs with a quantum yield of ~7.0% and a lifetime of ~9.5 ns using a multi-stimulus responsive IDP Rec1-resilin. In this process, Rec1-resilin acts concurrently as the structure directing agent, the reducer, and the stabilizer once Pt-NMQC is being formed. The in-depth analyses of the SAXS data and modeling confirmed that after interaction and stabilization of Pt-NMQC the overall structural ensemble of Rec1-resilin remained intrinsically disordered; however, reorganization of molecular conformation from a partially collapsed Gaussian chain IDP towards a swollen coil Gaussian chain IDP was observed. We showed spectroscopically that electronic energy transfer in close-packed Pt-NMQCs solids arises from dipole–dipole inter-dot interactions between proximal dots, which can be attributed to fluorescence resonance energy transfer (FRET) between two different sized QDs. This work highlights the application of an IDP as a multifunctional template, and the method applied in this work can be generally extended to generate engineered sub-nanoparticles of other NMQCs, such as silver, copper, palladium, and their alloys. The resilin-directed sub-nanoclusters can be potentially applied as multifunctional nanoconjugates in the field of biosensors, bioimaging, and catalysis, owing to the unusual multi-stimuli responsiveness, non-specific interactions, and conformational dynamics of the IDP.

## Supplementary Materials

The following are available online at https://www.mdpi.com/2079-6374/9/4/128/s1, Figure S1: Structural consensus and amino acid sequence in Rec1-resilin, Figure S2: MALDI-TOF mass spectra of synthesized Rec1-resilin, Figure S3: Effect of SAXS radiation time on scattering pattern of blue fluorescent Pt-NMQCs-Rec1-resilin nanobioconjugates, Figure S4: 3D-fluorescence contour plot of pristine Rec1-resilin at pH >10.5, Figure S5: Pt 4f XPS spectrum and peak fit of blue fluorescent Pt-NMQCs-Rec1-resilin nanobioconjugates, Figure S6: Fluorescence emission spectra of Pt-NMQCs-Rec1-resilin nanobioconjugates measured as a function of Pt concentration at different excitation wavelength, Figure S7: (A) Dityrosine fluorescence peak position and intensity of Rec1-resilin at different Pt concentration. (B) Dityrosine fluorescence intensity ratio of Pt-NMQCs-Rec1-resilin nanobioconjugates to Rec1-resilin at different Pt concentration, Figure S8: Photograph of green fluorescent Pt-NMQCs-Rec1-resilin nanobioconjugates under 365 nm UV light after storage at ambient temperature for almost a year.
