# Supplementary material for: A Sustainable Biomineralization Approach for the Synthesis of Highly Fluorescent Ultra-Small Pt Nanoclusters
Source: Biosensors (Basel). 2019 Oct 29;9(4):128. doi: 10.3390/bios9040128 (PMC6956208; doi:10.3390/bios9040128)
Supplement: Supplementary file 1 [file biosensors-09-00128-s001.pdf]

# Supplementary Materials

*Article*

# A Sustainable Biomineralization Approach for the Synthesis of Highly Fluorescent Ultra-Small Pt Nanoclusters

**Rajkamal Balu [1], Robert Knott [2], Christopher M. Elvin [3], Anita J. Hill [4], Namita R. Choudhury [1,*] and Naba K. Dutta [1,*]**

[1] Chemical and Environment Engineering, School of Engineering, RMIT University, Melbourne, VIC 3000, Australia.
[2] Australian Centre for Neutron Scattering (ACNS), Australian Nuclear Science and Technology Organisation (ANSTO), Lucas Heights, NSW 2234, Australia.
[3] CSIRO Agriculture, Level 6, Queensland Bioscience Precinct, St Lucia, QLD 4067, Australia.
[4] CSIRO Manufacturing, Bayview Ave, Clayton, VIC 3168, Australia.
* Correspondence: namita.choudhury@rmit.edu.au (N.R.C.); naba.dutta@rmit.edu.au (N.K.D.)

Received: 14 August 2019; Accepted: 21 October 2019; Published: 29 October 2019

```
Consensus   GGRPSDSYGAPGGGN
MHHHHHHPEP  PVNSYLP
               PSDSYGAPGQSGP
            GGRPSDSYGAPGGGN
            GGRPSDSYGAPGQGQGQGQGQGGYAGK
               PSDSYGAPGGGNGN
            GGRPSSSYGAPGGGN
            GGRPSDTYGAPGGGN
            GGRPSDTYGAPGGGGNGN
            GGRPSSSYGAPGQGQGNGN
            GGRPSSSYGAPGSGN
            GGRPSDTYGAPGGGN
            GGRPSDTYGAPGGGNN
            GGRPSSSYGAPGGGN
            GGRPSDTYGAPGGGNGNGS
            GGRPSSSYGAPGQGQGGF
            GGRPSDSYGAPGQNQK
               PSDSYGAPGSGNGN
            GGRPSSSYGAPGSGP
            GGRPSDSYGPPASG
```

**Figure S1.** Structural consensus and alignment of amino acid (single-letter code) repeat sequence in Rec1-resilin.

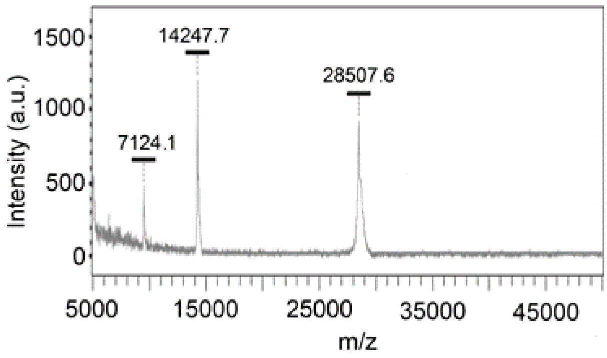


**Figure S2.** MALDI-TOF mass spectra of synthesized Rec1-resilin. The three m/z species (left to right) detected are the $[M+H]^{+}$, $[M+2H]^{2+}$ and $[M+3H]^{3+}$ species of the protein.

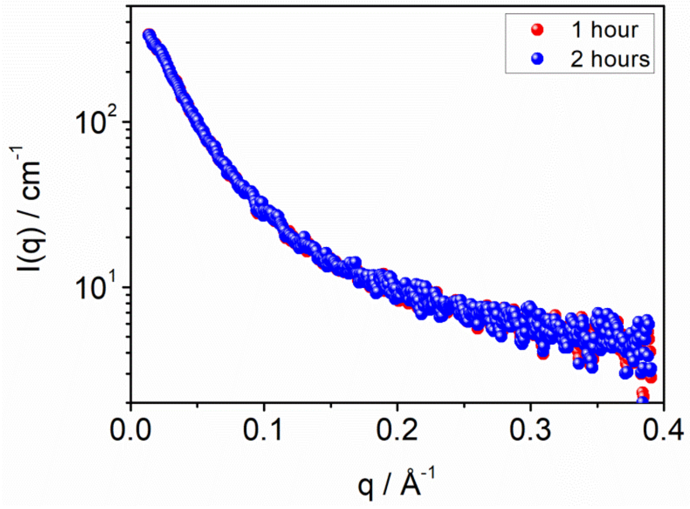


**Figure S3.** Effect of SAXS radiation time on scattering pattern of blue fluorescent Pt-NMQCs-Rec1-resilin nanobioconjugates.

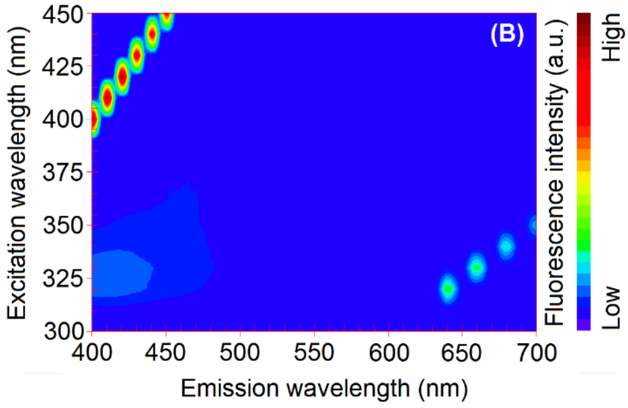


**Figure S4.** 3D-fluorescence contour plot of pristine Rec1-resilin (at pH > 10.5) equilibrated at 50 °C for 10 days.

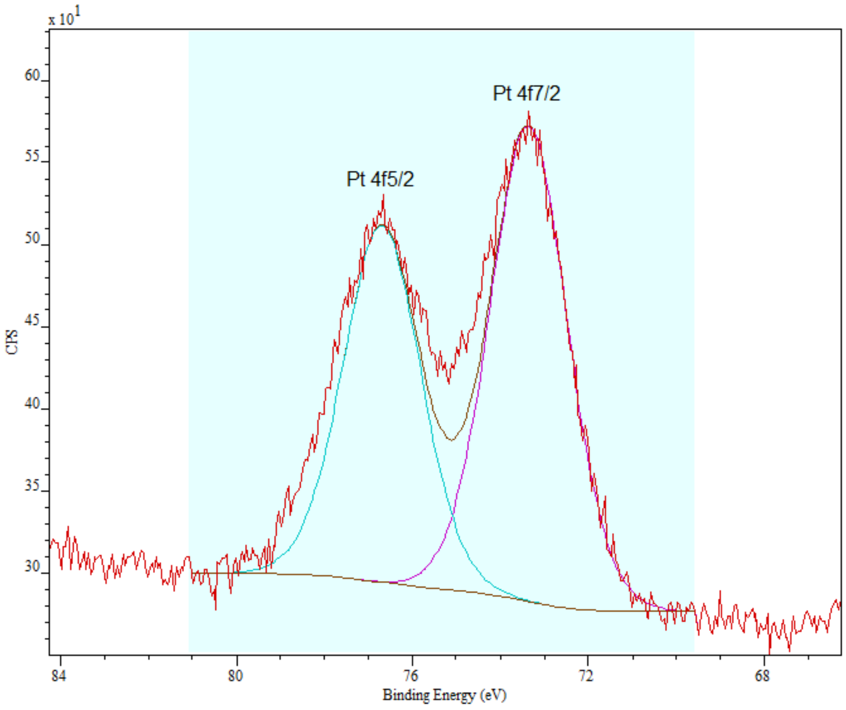


**Figure S5.** Pt 4f XPS spectrum and unimodel peak fit of blue fluorescent Pt-NMQCs-Rec1-resilin nanobioconjugates.

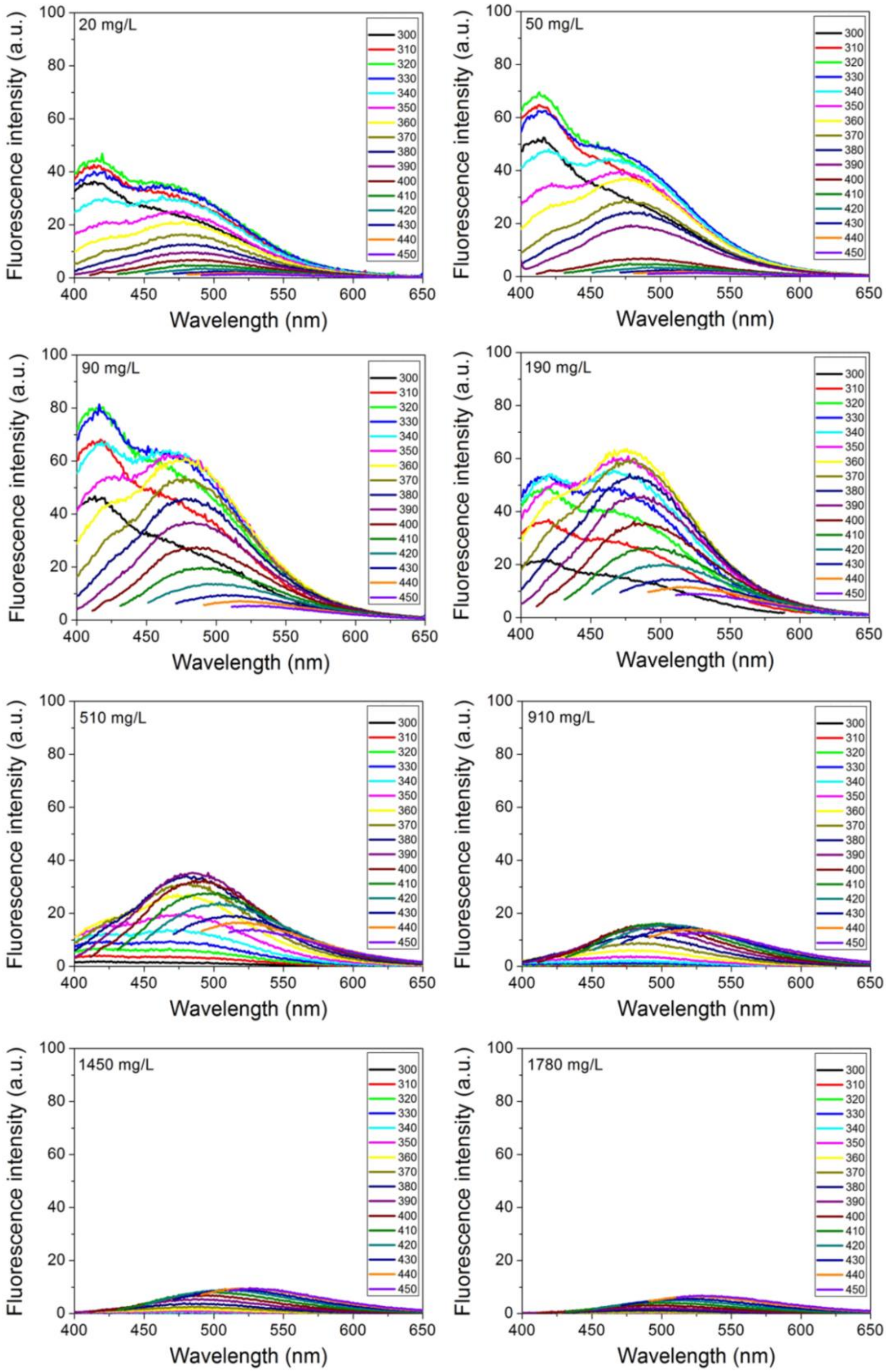


**Figure S6.** Fluorescence emission spectra of Pt-NMQCs-Rec1-resilin nanobioconjugates measured as a function of Pt concentration at different excitation wavelength (colour legends).

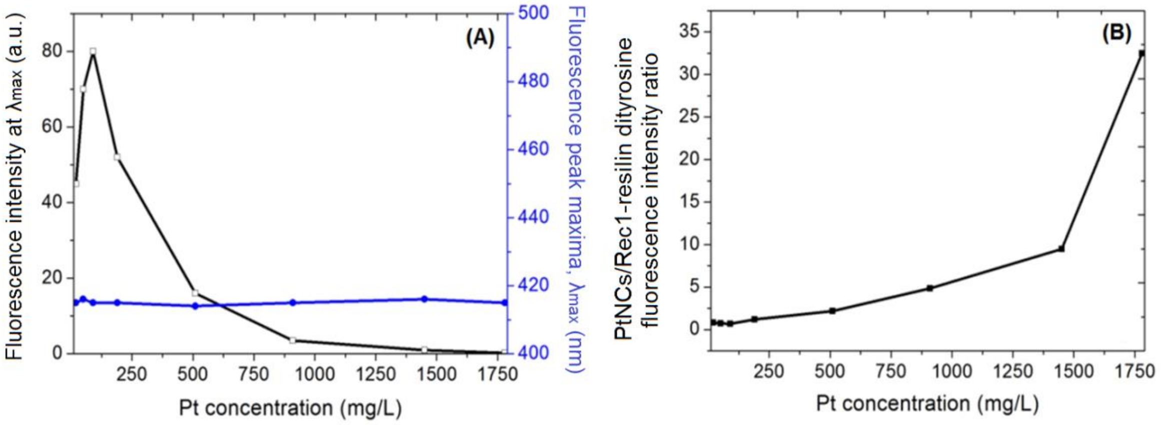


**Figure S7.** (A) Dityrosine fluorescence peak position (blue) and intensity (black) of Rec1-resilin at different Pt concentration. (B) Dityrosine fluorescence intensity ratio of Pt-NMQCs-Rec1-resilin nanobioconjugates to Rec1-resilin at different Pt concentration.

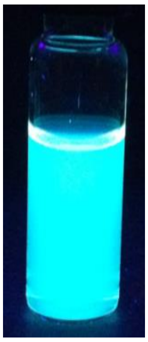

**Figure S8.** Photograph of green fluorescent Pt-NMQCs-Rec1-resilin nanobioconjugate dispersion under 365 nm UV light after storage at ambient temperature for almost a year.
